# Global illumination rendering versus volume rendering for the forensic evaluation of stab wounds using computed tomography

**DOI:** 10.1038/s41598-022-06541-9

**Published:** 2022-02-14

**Authors:** Wataru Fukumoto, Nobuo Kitera, Hidenori Mitani, Takahiro Sueoka, Shota Kondo, Ikuo Kawashita, Yuko Nakamura, Masataka Nagao, Kazuo Awai

**Affiliations:** 1 Department of Diagnostic Radiology, Graduate School of Biomedical and Health Science, Hiroshima University, 1-2-3 Kasumi, Minamiku, Hiroshima 734-8551 Japan; 2 Center for Cause of Death Investigation Research, Graduate School of Biomedical and Health Science, Hiroshima University, 1-2-3 Kasumi, Minamiku, Hiroshima 734-8551 Japan; 3 Department of Diagnostic Radiology, Hiroshima University Hospital, 1-2-3 Kasumi, Minamiku, Hiroshima 734-8551 Japan

**Keywords:** Biotechnology, Computational biology and bioinformatics, Medical research

## Abstract

We compared three-dimensional (3D) CT images of stabbing victims subjected to volume-rendering (VR) or global illumination-rendering (GIR), a new technique now available for the reconstruction of 3D CT images. It simulates the complete interactions of photons with the scanned object, thereby providing photorealistic images. The diagnostic value of the images was also compared with that of macroscopic photographs. We used postmortem 3D CT images of 14 stabbing victims who had undergone autopsy and CT studies. The 3D CT images were subjected to GIR or VR and the 3D effect and the smoothness of the skin surface were graded on a 5-point scale. We also compared the 3D CT images of 37 stab wounds with macroscopic photographs. The maximum diameter of the wounds was measured on VR and GIR images and compared with the diameter recorded at autopsy. The overall image-quality scores and the ability to assess the stab wounds were significantly better on GIR than VR images (median scores: VR = 3 vs GIR = 4, p < 0.01). The mean difference between the wound diameter measured on VR and GIR images and at autopsy were both 0.2 cm, respectively. For the assessment of stab wounds, 3D CT images subjected to GIR were superior to VR images. The diagnostic value of 3D CT GIR image was comparable to that of macroscopic photographs.

## Introduction

Post-mortem CT (PMCT) is a supplementary diagnostic modality available to forensic pathologists and coroners investigating the cause of death^1–4^. As PMCT images are occasionally used by non-experts in radiology, they must be easily interpretable. The three-dimensional (3D) reconstruction is one of the most interpretable presentation format of CT images and the 3D CT images facilitate explaining forensic radiologic findings including bone fractures and injuries to non-experts in radiology^5–7^. However, to date, few studies have addressed the diagnostic value of 3D CT images in the forensic field.

The global illumination rendering (GIR) technique, widely used by the film industry, video games, lighting engineering, and flight simulators, is now available to reconstruct 3D CT images in the medical field^3,6–14^. Because it can simulate complete interactions of photons with the scanned object, thereby providing photorealistic images and improving the visualization of fine details, it is superior to the conventional volume rendering (VR) technique^6,7,9^.

PMCT has proven to be useful for the depth assessment of penetrating deep organ injuries such as pneumothorax, intestinal perforation, and intra-abdominal bleeding due to stabbing^15,16^. Forensically, the careful inspection of stab wounds is necessary to determine the type of weapon, its direction, and the applied force^17^. However, forensic assessment on routine 2D axial CT images can be difficult. The visualization and detection of stab wounds may be improved on 3D CT images with GIR technique because depth perception and the shadowing effects of subtle superficial findings are enhanced^9^.

Under the hypothesis that the diagnostic value of postmortem 3D images subjected to GIR and of macroscopic photographs is comparable and that GIR helps in the investigation of stab wounds on the body surface, we evaluated their diagnostic utility.

## Materials and methods

This retrospective study was approved and prior informed consent was waived by Ethical Committee for Epidemiology of Hiroshima University. The study is in accordance with relevant guidelines and regulations.

### Study subjects

We enrolled 14 stabbing victims (8 males, 6 females; median age 42 years, range 3–89 years) who had undergone autopsy and CT studies between September 2018 and March 2021 in our center for cause of death investigation research. Their median height was 164.5 cm (range 96.0–178.0 cm), their median weight was 53.7 kg (range 12.2–89.0 kg).

Autopsy revealed 104 stab wounds in the 14 victims. Of the wounds, 43 were on the front of the body, 48 on the back, and 13 were on the side. A knife was used to kill 13 victims, a razor to kill one.

The median interval between death and the acquisition of CT scans was 3 days (range 1–12 days).

### CT scans

The 14 victims were scanned in body bags with a 16-row multi-detector CT scanner (Aquilion Lightning; Canon Medical Systems). Helical scans were acquired at a tube voltage of 120 kV; the tube current was regulated by automatic exposure control with a preset noise level of 8 Hounsfield units. The other scanning parameters were rotation time 0.75 s, beam collimation 0.5 × 16 mm, helical pitch 0.98, and display field-of-view 50 × 50 cm^2^.

Using axial CT images (slice thickness 1.0 mm), a medical radiographer (N.K.) with 9 years of experience reconstructed the 3D CT images with VR and GIR on a workstation (Vitrea, Canon Medical Systems). The virtual light source, using default settings, was at the front (VR image) and at the upper right (GIR images). Opacity was set at a window width/level operation of 600/0 Hounsfield Unit (HU) for VR images and at 900/100 HU for GIR images, for the clear visualization of the stab wounds. Color adjustments were made so that the VR and GIR images were the same.

### Image analysis

After comparing the 3D CT GIR images with conventional 3D CT-VR images, 3 diagnostic radiologists (S.K., T.S. and H.M.) with 7 or more years of experience compared their overall image quality for the 3D effect and the smoothness of the skin surface. They independently scored the VR and GIR images of each victim using a 5-point Likert scale where 1 = unacceptable image quality (the image was only slightly 3D and the surface was irregular), 2 = suboptimal (the image was only slightly 3D and the skin-surface pattern was remarkably striped, 3 = acceptable (the image was 3D and the skin surface was slightly striped, 4 = better than average (the image was almost completely 3D and the skin surface was smooth), and 5 = excellent (the image was completely 3D , shading was adequate, and the skin surface was smooth)^18^.

Then a board-certified radiologist (W.F. with 3 years of experience with PMCT), a forensic pathologist (M.N. with 35 years of experience in forensic autopsies, and a coroner (T.M. with 27 years of experience with postmortem inspections) inspected the images. They graded visualization of the stab wounds on the 3D CT images on a 5-point Likert scale where 1 = undetectable (wound detection was difficult), 2 = suboptimal (detailed wound assessment was difficult), 3 = acceptable (wound assessment possible but inferior to macroscopic photographs), 4 = better than average (detailed wound assessment was as on macroscopic photographs.) and 5 = excellent (detailed wound assessment better than on macroscopic photographs)^18^.

In their wound assessments, the readers inspected 43 wounds on the front of the body because on 3D CT images subjected to VR or GIR, stab wounds on the back and side were masked by the body bag, the CT bed, and the victims’ arms. Of these 43 wounds, 6 were excluded because the knife was stuck in the wound (n = 1), the wounds resulted in bowel prolapse (n = 2), the wounds were covered with gauze (n = 3). Consequently, the readers assessed 37 stab wounds on VR and GIR images.

The radiologist (W.F.) recorded the maximum diameter of these stab wounds on VR and GIR images and compared it with the diameter measured at autopsy.

### Statistical analysis

Differences between the image-quality score of VR and GIR images were analyzed with the two-sided Wilcoxon signed-rank test.

Bland–Altman analysis was used to identify the difference between the diameter measured on 3D CT image and at autopsy.

Differences of p < 0.05 were considered statistically significant.

## Results

### Overall image-quality of VR and GIR images

**Figure 1:**
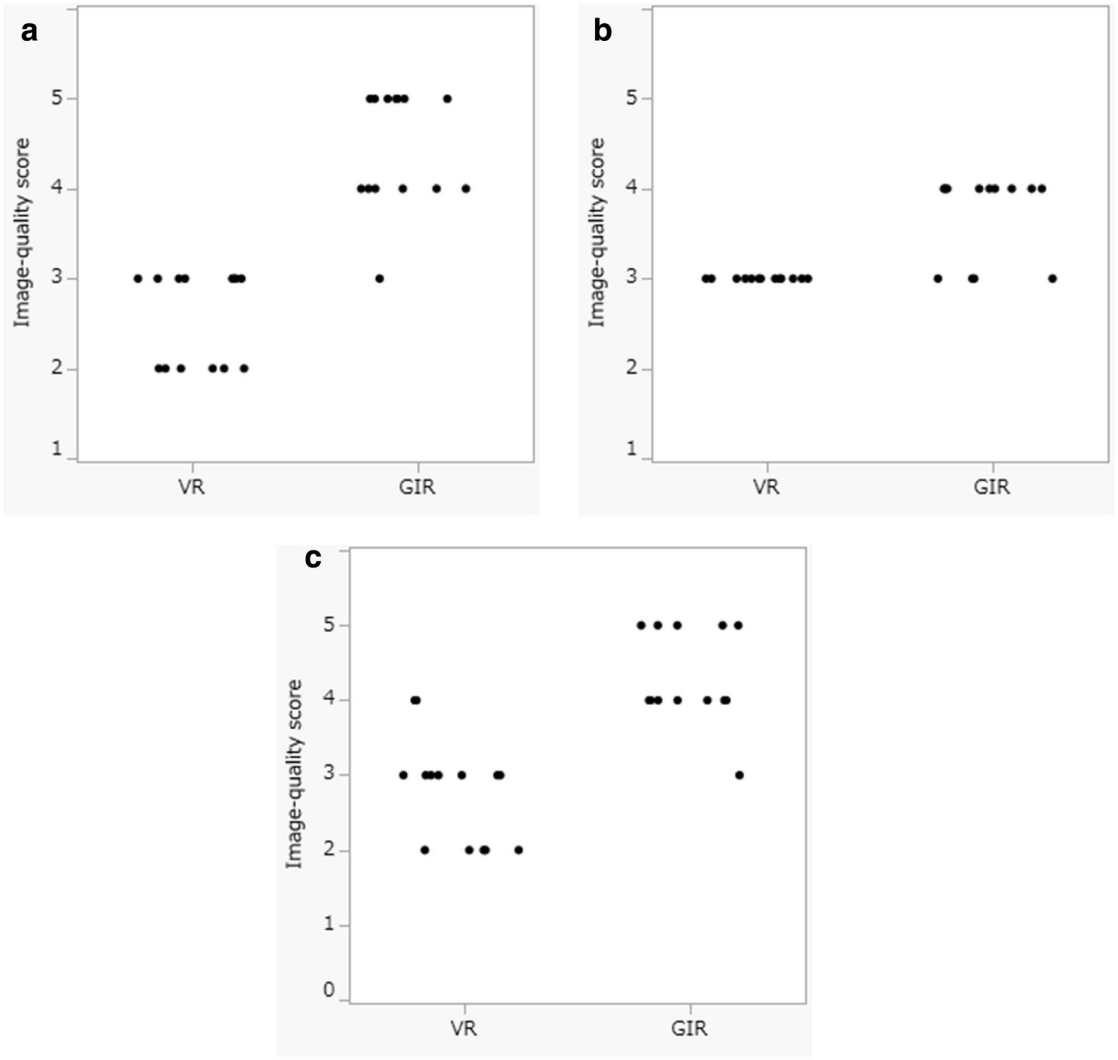
Overall image-quality scores of VR and GIR images of 14 stabbing victims. ((**a**) Radiologist 1, (**b**) Radiologist 2, (**c**) Radiologist 3). The image-quality scores for the 3D effect and for smoothness of the skin surface were significantly higher on 3D CT GIR than 3D CT VR images (median image-quality scores: VR = 3 vs GIR = 4, p < 0.01).

**Figure 2:**
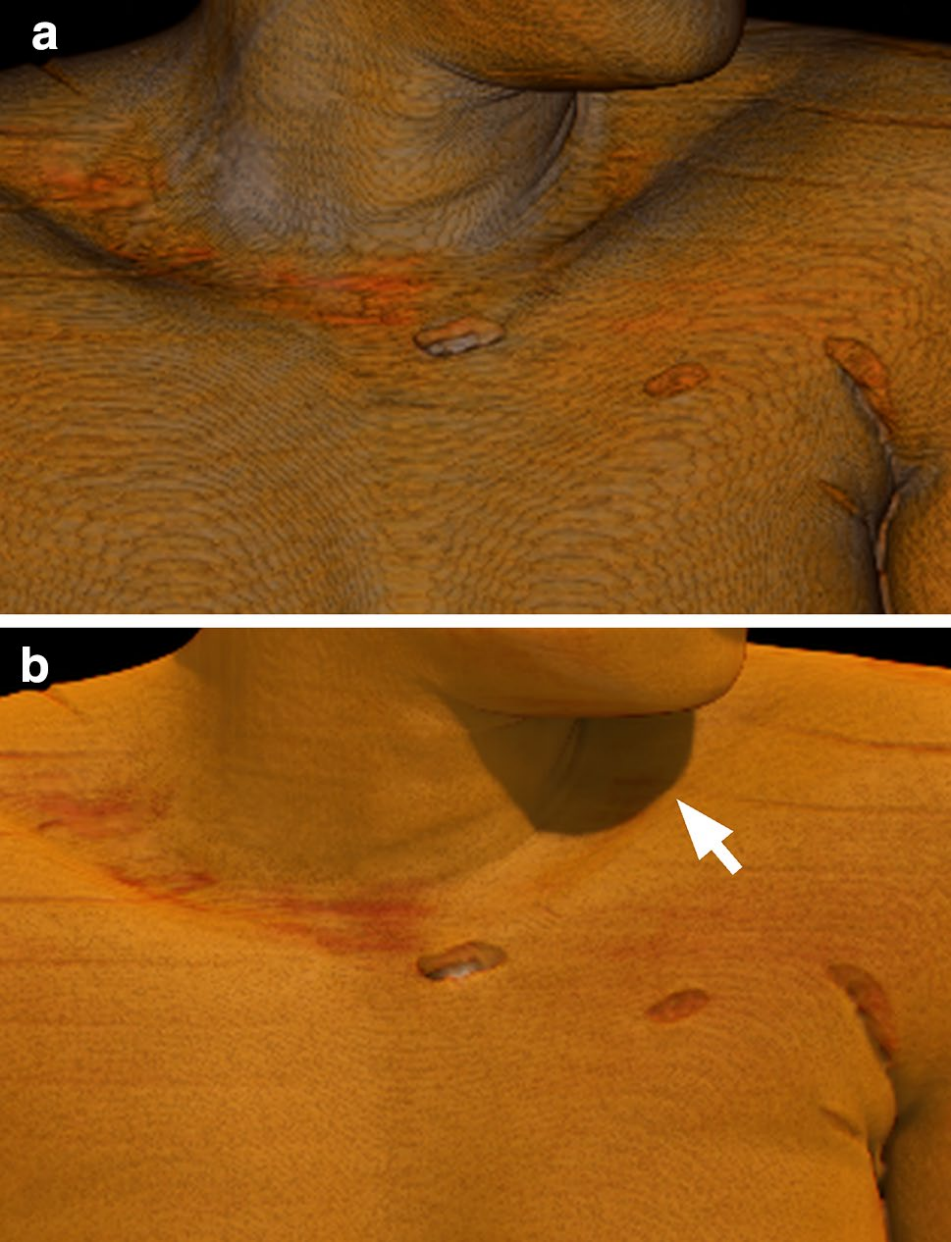
A 36 year-old woman killed by multiple stabs with a knife. ((**a**) 3D CT VR image, (**b**) 3D CT GIR image). (**a**) 3D CT VR images showed remarkable striping reminiscent of contour lines on the skin surface. (**b**) GIR applies a complex lighting model and simulates light sources from different angles, thereby improving the 3D effect and smoothing the skin surface. The shadow under the chin is clearly visualized (arrow).

The image-quality scores assigned by the 3 diagnostic radiologists to the VR and GIR images of the 14 stabbing victims are shown in Fig. 1. They were significantly higher for GIR images with respect to the 3D effect and the smoothness of the skin surface (median image-quality score: VR = 3 vs GIR = 4, p < 0.01). The GIR images were of greater 3D appearance because shadowing was appropriate and the stripes reminiscent of contour lines were suppressed, resulting in a smoother skin surface (Fig. 2).

### Stab wound assessment on 3D CT images

**Figure 3:**
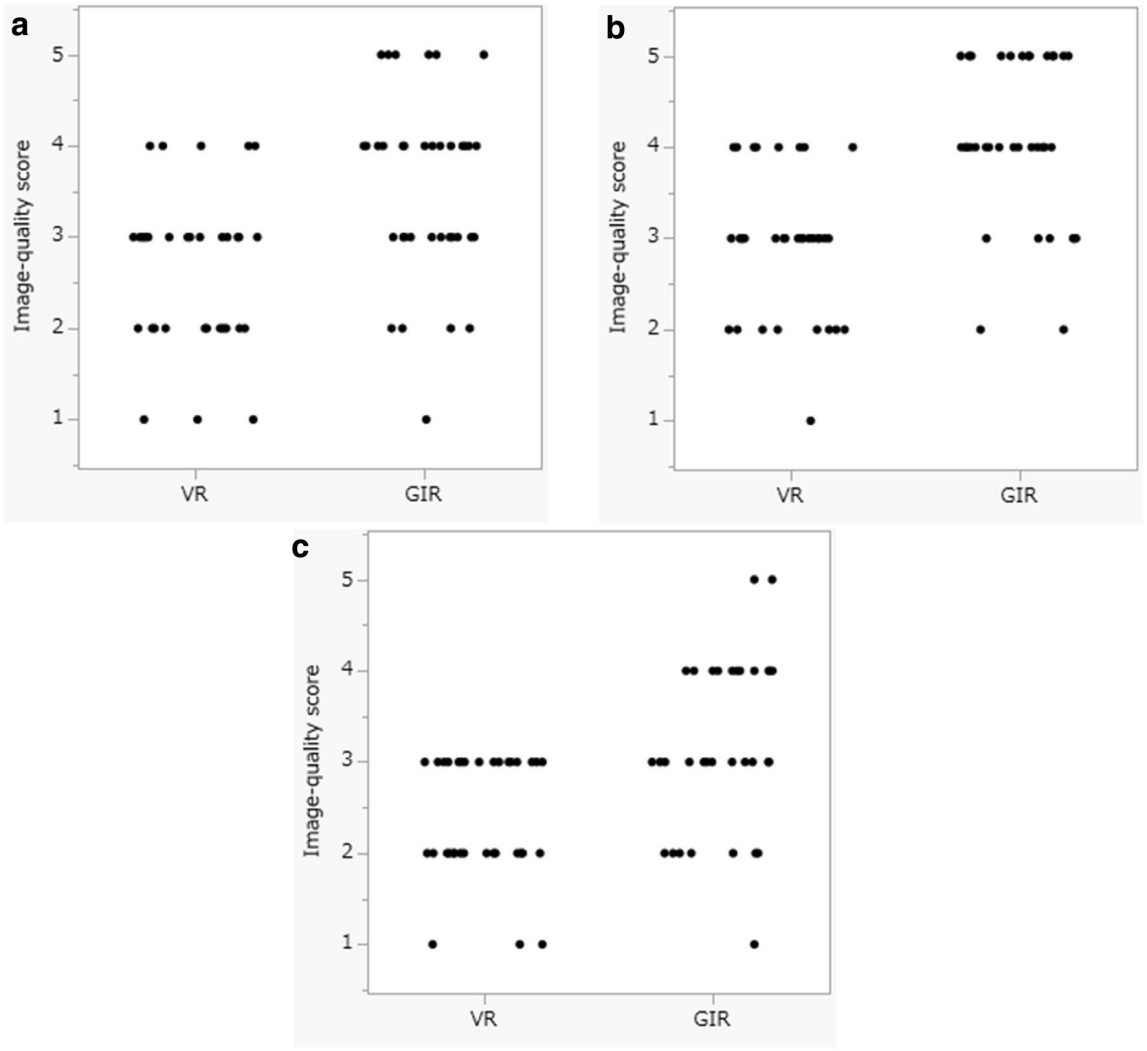
Image-quality scores assigned for 3D CT GIR and 3D CT GIR images by 3 observers. ((**a**) Radiologist, (**b**) forensic pathologist, (**c**) coroner). The scores for wound-margin assessment, the wound shape, and the wound depth perception were significantly higher for 3D CT GIR than 3D CT VR images (median scores VR = 3 vs GIR = 4, p < 0.01).

**Figure 4:**
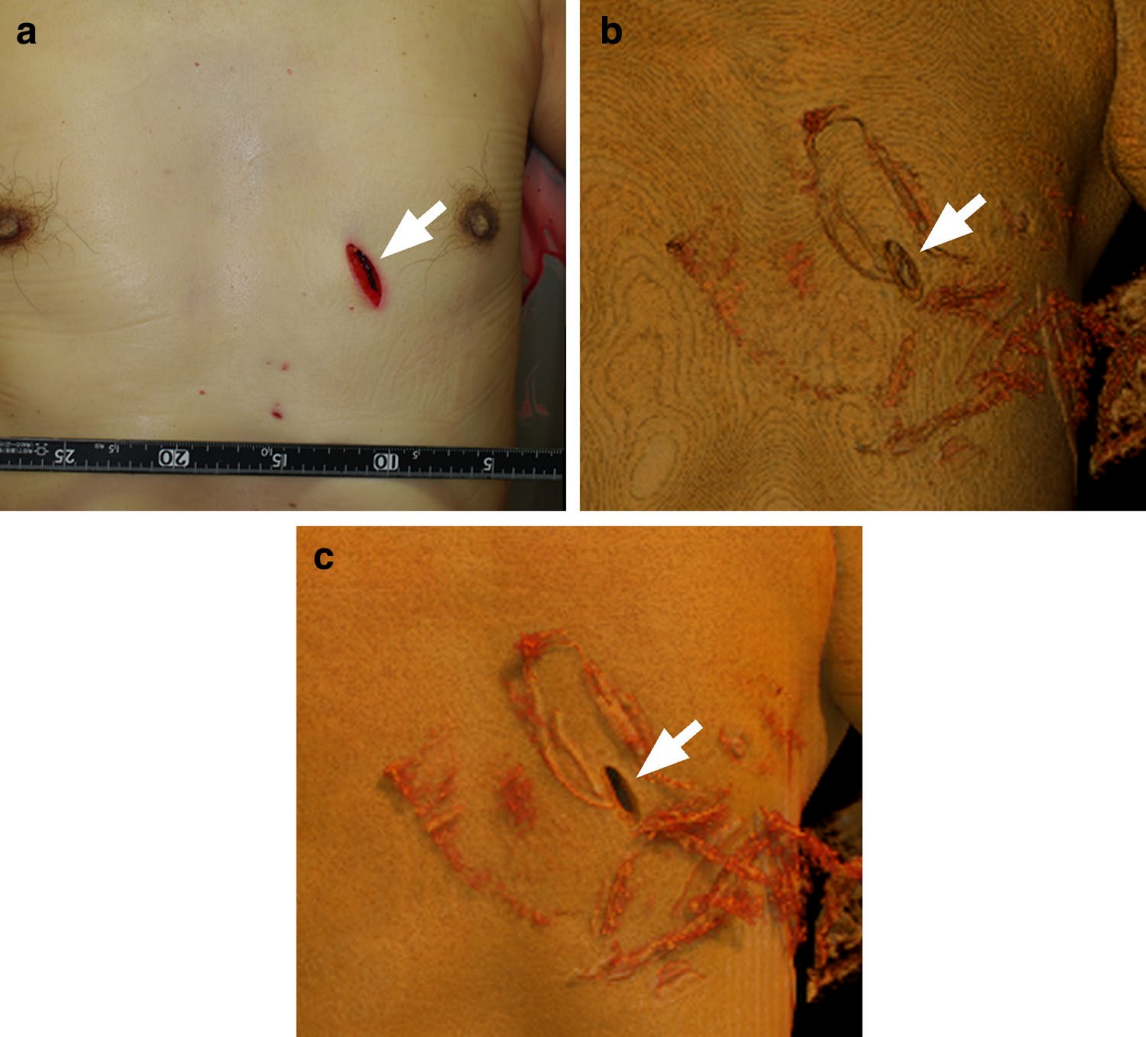
A 62-year old man killed with a knife. ((**a**) Photograph, (**b**) 3D CT VR image, (**c**) 3D CT GIR image). GIR improved the depth perception of the stab wound (arrow). The images were photorealistic and improved visualization of fine details including the wound margin.

The image-quality scores of the stab wounds on VR and GIR images, graded by the radiologist, forensic pathologist, and coroner, were significantly higher for GIR than VR images with respect to the wound margin, shape, and the depth assessment (median quality score: VR = 3 vs GIR = 4, p < 0.01) (Figs. 3, 4).

**Figure 5:**
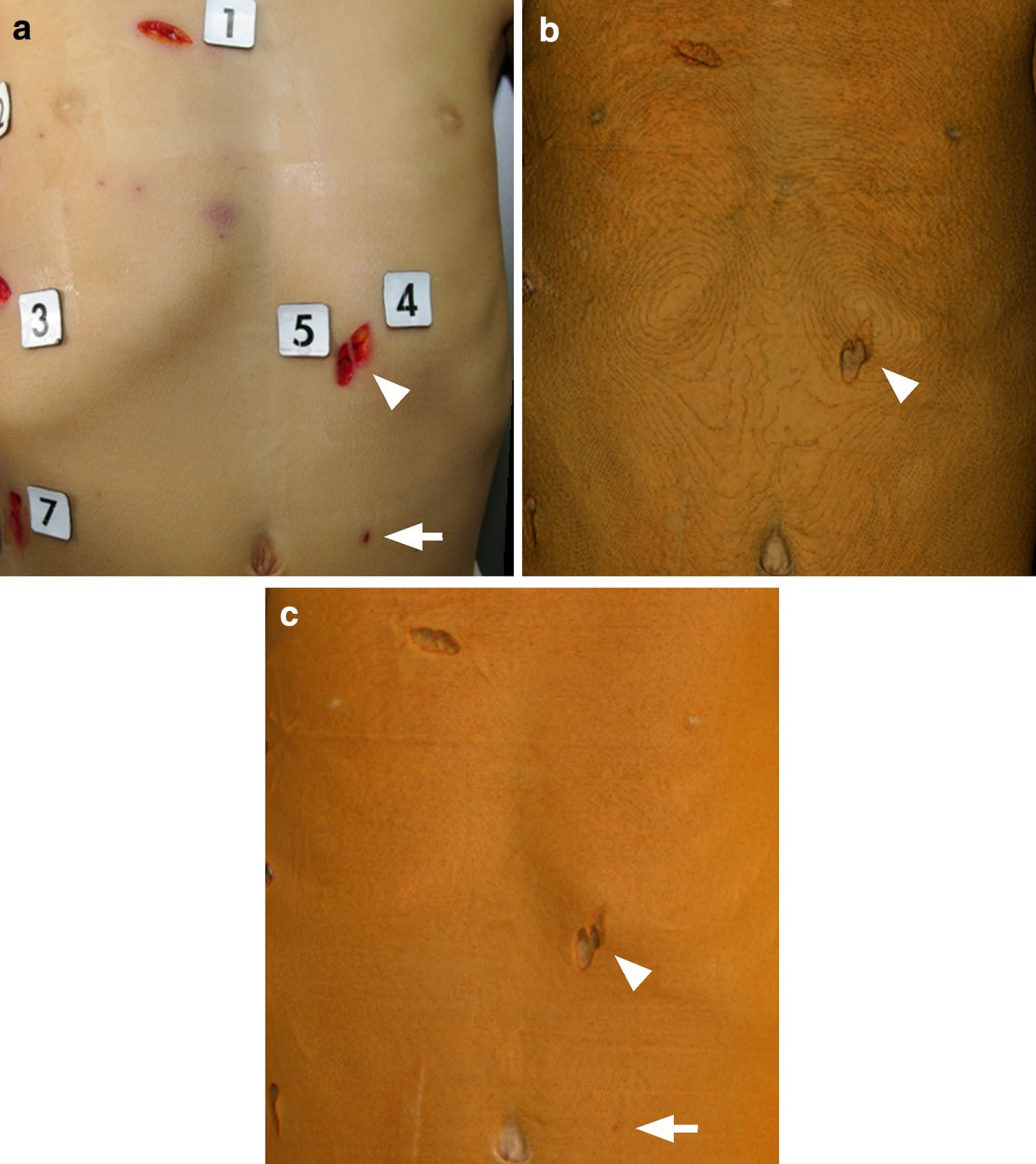
A 5-year-old boy killed by multiple stabs with a knife. ((**a**) Photograph, (**b**) 3D CT VR image, (**c**) 3D CT GIR image). The small, shallow stab wound was not detectable on the 3D CT VR image; it was observable on the 3D CT GIR image (arrow). VR failed to distinguish between 2 adjacent stab wounds. On the 3D CT GIR image wound details are observable (arrow head).

Of the 37 inspected stab wounds, 3 were not detectable on VR images because they were small and shallow; 2 of these were detectable on GIR images (Fig. 5).

The diagnostic value of 3D CT GIR images and macroscopic photographs was comparable.

### Maximum diameter of the stab wounds

At autopsy, the median maximum diameter of the 37 stab wounds on the front of the body was recorded as 2.6 cm (range 0.1–8.0 cm). After excluding the 3 wounds not visualized on VR images, the median maximum diameter of the remaining stab wounds was 2.7 cm (range 0.5–8.0 cm). The median maximum diameter calculated on VR images was 2.4 cm (range 0.6–7.2 cm), on GIR images it was 2.4 cm (range 0.7–7.7 cm).

Bland–Altman analysis indicated that the mean difference in the wound diameter on VR and GIR images vs the diameter recorded at autopsy was 0.2 ± 0.1 cm; the limit of agreement was − 0.7 and 1.2 for VR and GIR images, respectively.

## Discussion

### Comparison of 3D CT VR and GIR images

The 3D effect, the smoothness of the skin surface, and for a detailed assessment of stab wounds, GIR images were superior. We found that the diagnostic value of 3D CT GIR image was similar to macroscopic photographs.

As GIR technique simulates the propagation and interaction of light rays as they pass through volumetric data^3,6–14^, it improves the 3D effect, depth perception, and shows subtle irregularities of the skin surface. VR, on the other hand, applies a local lighting model to volumetric data^12^. Among 3 small, shallow stab wounds that were not detectable on VR images, 2 were displayed on GIR images. VR also failed to distinguish between two adjacent stab wounds, GIR did not (see Fig. 3). Our findings indicate that GIR yields a more photorealistic representation of 3D images than the conventional VR technique.

Although the utility of GIR application to 3D CT in the medical field has been reported^5–9^, only few studies assessed the diagnostic value of GIR technique^6^. Ours is the first documentation that GIR is superior to conventional VR at the postmortem investigation of stabbing victims.

### Advantage of GIR in the assessment of stab wounds

The detailed inspection of stab wounds is crucial for the identification of the stabbing instrument, the applied force, the stab trajectory of the weapon, and the position of the victim and perpetrator at the time of the assault^17^. The application of GIR to 3D CT imaging facilitates the detailed assessment of stab wounds and yields images that are comparable to macroscopic photographs.

For the assessment of stab wounds, 3D CT images have some advantages over macroscopic photographs. They can be obtained quickly because the CT instrument can scan the entire body in several tens of seconds while it takes more time to acquire photographs and their quality depends on the photographer and the light conditions. Although advanced 3D surface imaging techniques using a multi-camera rig have been introduced to reduce the acquisition time, these systems are not yet widely used^19^.

**Figure 6:**
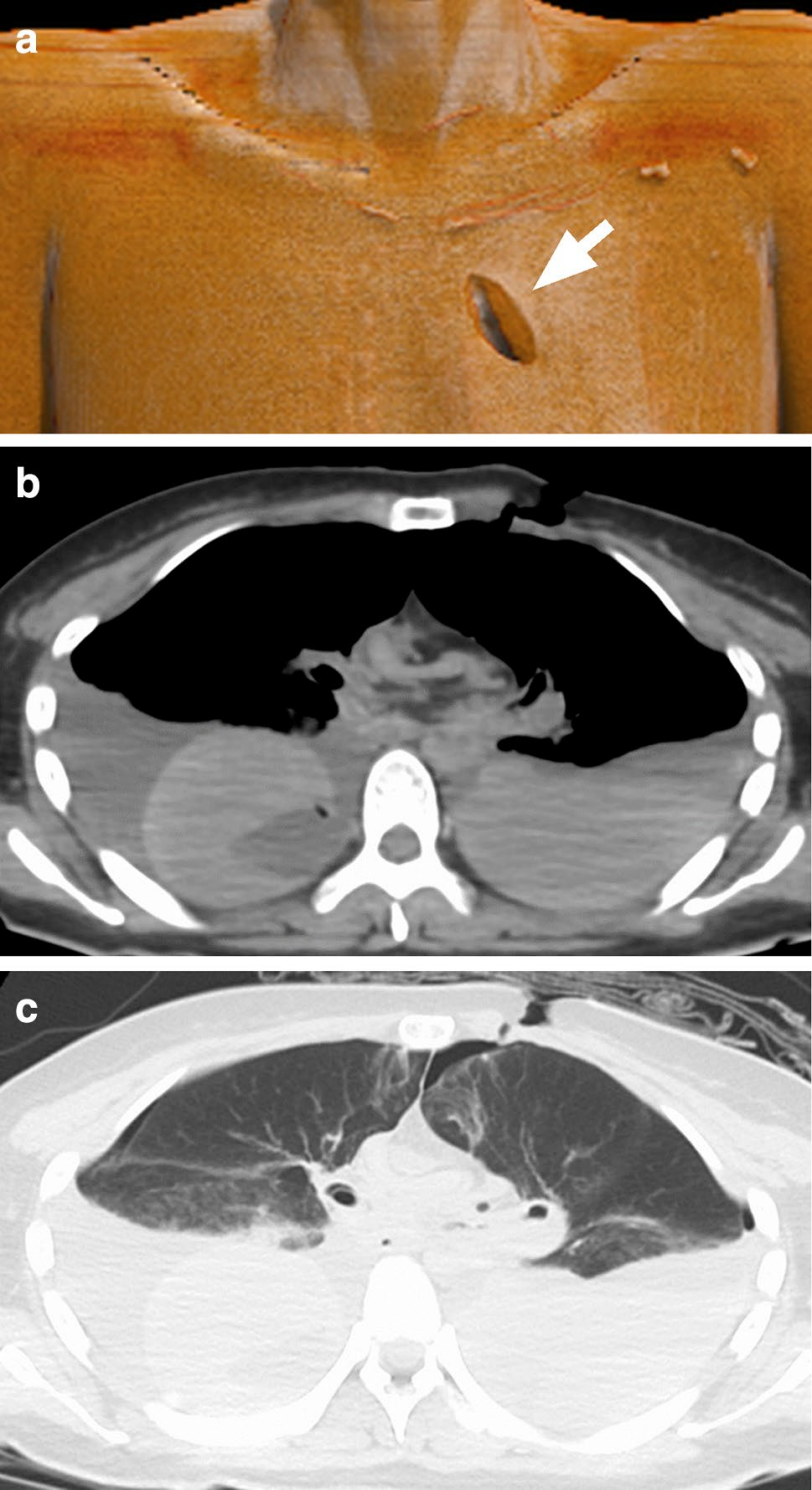
A 49-year-old woman killed with a knife. ((**a**) 3D CT GIR image, (**b**) axial CT image (soft kernel), (**c**) axial CT image (lung kernel)). (**a**) 3D CT GIR image showing a stab wound at the left anterior thorax (arrow). (**b,c**) The axial CT images show bilateral hemopneumothorax suggesting that the knife stabbed into the left anterior thorax penetrated the mediastinum and reached the right lung.

With 3D CT imaging the forensic findings on axial images can be easily explained to non-radiologists^5–7^. Besides, the wound depth and the direction of the stab can be ascertained by confirming injuries and hemorrhages below the skin surface by axial CT images (see Fig. 6). Lastly, post-traumatic stress disorder for persons attending the trial is one of the most important issue in miserable case because they are forced directly to look at gruesome photographs^20,21^. The postmortem 3D CT image which is adjusted color tone may be effective to spare PTSD for them.

On the other hand, macroscopic photographs have a significant advantage, i.e. they provide the color information important for the accurate assessment of the wound edges. Measurements on the corpse and photographs providing color information are indispensable for the primary evaluation. Since CT images and macroscopic photographs each have advantages, we should use them according to a purpose for forensic investigations.

### Measurement of the stab-wound diameter on 3D CT images

The mean difference between the diameter of stab wounds recorded at autopsy and on 3D CT VR and GIR images was 0.2 cm; GIR failed to lower the difference. The quality of 3D CT images is highly dependent on the quality of the original dataset^7^. To improve the quantitative assessment of 3D CT images, ultra-high resolution CT scanning may be required because its special resolution is superior to conventional CT^22–25^. In addition, in order to improve the quality of 3D CT images, the wound should not be masked when CT is scanned. The wounds on the back or side may be masked due to a body bag, CT bed and their arms. Keeping the stab wound on the back away from the CT bed with a sponge or cushion, or raising the arm, is effective in improving the image quality of 3D CT images. Placing additional external markers (radiopaque rings) may help to detect stab wounds on the back or side of the victim’s body^26^.

### Limitations

Our study has some limitations. The readers’ judgement that GIR were superior to VR images was subjective because there was no objective assessment way for 3D CT images available. Also, the study population was small and we did not assess stab wounds delivered by devices other than knives. Differences in minor settings, including the placement of the virtual light, the opacity, and the type of workstation used may have slightly affected the quality of VR and GIR images.

## Conclusion

For the investigation of stab wounds, 3D CT images subjected to GIR were superior to 3D CT VR images. The diagnostic quality of 3D CT GIR images was comparable to macroscopic photographs.

## Abbreviations

3D: Three-dimensional
VR: Volume-rendering
GIR: Global illumination-rendering
PMCT: Postmortem computed tomography
PTSD: Post-traumatic stress disorder
